# Exploring the association between extra-cardiac troponin elevations and risk of future mortality

**DOI:** 10.5937/jomb0-25262

**Published:** 2020-10-02

**Authors:** Giuseppe Lippi, Fabian Sanchis-Gomar

**Affiliations:** 1 University of Verona, University Hospital of Verona, Section of Clinical Biochemistry, Verona, Italy; 2 University of Valencia, Faculty of Medicine, Department of Physiology, Valencia, Spain; 3 INCLIVA Biomedical Research Institute, Valencia, Spain

**Keywords:** mortality, myocardial infarction, cardiac injury, troponin, troponin, srčano oštećenje, infarkt miokarda, mortalitet

## Abstract

Although the measurement of cardiac troponin I (cTnI) and T (cTnT) has now become the cornerstone for diagnosing cardiac injury, both ischemic and non-ischemic, recent evidence has become available that many patients display extra-cardiac causes of cTn elevations and carry a considerably enhanced risk of future mortality. The current literature data suggests that cTn elevations may be equally common in patients with cardiac and extra-cardiac diseases. Among the latter cohort of patients, the leading extra-cardiac diseases which may be responsible for either cTnI or cTnT elevations include infectious diseases/sepsis, pulmonary disorders, renal failure, malignancy, as well as gastrointestinal, neurological and musculoskeletal diseases. What also emerges rather clearly from the current literature data, is that the risk of dying for extra-cardiac diseases is higher (i.e., between two to three-fold) in patients with extra-cardiac cTn elevations than in those with cardiac pathologies, and that the most frequent cause of death would then be infections/sepsis, followed by malignancy, respiratory disorders, myocardial infarction, gastrointestinal and neurological diseases, heart failure, stroke, cardiac arrhythmias, renal failure, psychiatric, metabolic, urogenital and musculoskeletal disorders. These figures would lead to conclude that there is a considerable risk that the underlying pathology causing cardiac injury and cTn elevation would then become the cause of death in these patients. This important evidence shall lead the way to defining appropriate and effective strategies for managing patients with extra-cardiac cTn elevations, so that their risk of future death could be prevented or limited.

## Abstract (sr)

Mada su merenja srčanog troponina I (cTnI) i T (cTnT) ključna za dijagnostikovanje srčanog oštećenja, kako ishemijskog tako i ne-ishemijskog poslednji dokazi ukazuju da se kod mnogih pacijenata javljaju ekstra-srčani uzroci povećanja CTn i dovode do značajno povećanog rizika od budućeg mortaliteta. Podaci iz trenutno dostupne literature ukazuju da povećanja cTn mogu da se podjednako pojave kod pacijenata sa srčanim i ekstra-srčanim oboljenjima. Kod poslednjih ispitivanih pacijenata vodeća ekstra-srčana oboljenja koja mogu biti odgovorna za povećanje cTnI ili cTnT su infektivna oboljenja/sepsa, plućni poremećaji, bubrežna oboljenja, maligna oboljenja, kao i gastrointestinalna, neurološka i mišićnoskeletna oboljenja. Prilično jasno trenutni literaturni podaci ukazuju da je rizik od umiranja od ekstra-srčanih oboljenja visok (npr. između dva do tri puta) u pacijenata sa ekstra-srčanim cTn povećanjima nego kod onih sa kardiološkom patologijom, i da najčešći uzrok smrti može biti infekcija/sepsa, praćena sa malignim, respiratornim poremećajima, infarktom miokarda, gastrointestinalnim i neurološkim oboljenjima, poremećajem rada srca, šlogom, srčanim aritmijama, bubrežnim poremećajem, psihijatrijskim, metaboličkim, urogenitalnim i mišićnoskeletnim poremećajima. Ovi podaci mogu da vode ka zaključku da postoji značajan rizik da druge patologije mogu dovesti do srčanog oštećenja i povećanja cTn te mogu biti i uzrok smrti ovih pacijenata. Ovaj značajni dokaz vodiće ka definisanju odgovarajuće i efektivne strategije za praćenje pacijenata sa povećanjem ekstra-srčanog cTn, kako bi rizik od buduće smrti bio preveniran ili umanjen.

## Introduction

The measurement of cardiac troponin I (cTnI) and T (cTnT) has now become the cornerstone for diagnosing cardiac injury, both ischemic and nonischemic [1], thus making the assessment of any other cardiac biomarker (e.g., creatine kinase isoenzyme MB or myoglobin) redundant and even obsolete [2]. Although both cTnI and cTnT were proven to be almost equally sensitive and specific for identifying cardiac injuries [3], convincing evidence now attests that the so-called high-sensitivity (HS) immunoassays are preferable over conventional (i.e., »contemporary sensitive«; CS) methods for routine assessment of cTns in clinical practice, because the former techniques would allow to detect a higher number of patients with primary or secondary cardiac damage [4], faster patient track [5], but may also enable a more accurate risk stratification of short-and longterm unfavorable outcome [6].

One of the most important consequences of introducing HS-cTn immunoassays in routine clinical practice is that the physiological concentrations of this biomarker (e.g., concentrations below the 99^th^ percentile upper reference limit (URL)) are now detectable in a vast number of ostensibly healthy people, varying between 50% and 95%. This ample range mostly depends on the analytical characteristics of the immunoassay (i.e., limit of detection and functional sensitivity), as well as on the target population [7] [8]. Concerning the latter aspect, some important physiological variable may generate a considerable impact on cTn values, especially including sex (i.e., cTn values are typically higher in men than in women), age (e.g., cTn values are higher in elderly people), body mass index (e.g., cTn values gradually increase in parallel with the body mass index), as well as cardiac volume (e.g., cTn values are correlated with the ventricular mass) [9] and physical activity [10]. Regardless of these biological determinants, it has now been unquestionably proven that the risk of future mortality is strongly associated with the baseline cTn value, whereby the death rate seemingly increases in parallel with the circulating cTn concentration [11] [12]. Notably, this important clinical association not only has been observed in patients with known cardiovascular disease (e.g., unstable angina, acute coronary syndrome (ACS), post-infarction heart failure, stroke), but also in those bearing a kaleidoscope of other extra-cardiac pathologies (e.g., sepsis, pulmonary thromboembolism, renal failure, surgery and so forth), as well as in the ostensibly healthy general population. The underlying reason has been recently postulated [13], and mostly encompasses a release of cTn from injured myocardium, which is strictly dependent on the cause and on the severity of cardiac injury. The most obvious questions at this point in time, which have not found definitive answers neither in a recent meta-analysis to the best of our knowledge [14], are (i) defining the most frequent causes of cTn elevations beside myocardial ischemia and/or injury, and (ii) identifying the leading causes of death in patients presenting with elevation of cTn values. The following part of this article is aimed to provide some reasonable answers to these clinically foremost questions.

## Causes of cTn elevation

As a rule of thumb, and recently confirmed by Giannitsis et al. [15], the re-expression of either cTnI or cTnT in pathologically regenerating skeletal muscle tissue has never been clearly demonstrated and the cross-reactivity of currently available immunoassays with skeletal cTns is highly unlikely (if not impossible), so that the most obvious source of measurable cTn is the heart tissue (i.e., the cardiomyocytes). Whatever elevation of cTn can now be measured in blood shall hence be sustained by a primary or secondary injury to cardiac tissue. Some interesting articles have been published on non-ischemic diseases associated with increased values of cTn, obviously measured with the new generation of HS cTn immunoassays.

The first study which addressed the issue of the leading determinants of HS cTn elevations in patients with extra-cardiac diseases has been published by Irfan et al., in 2012 [16]. The authors reviewed all the possible causes of cTnT elevation (i.e., value >99^th^ percentile URL of 14 ng/L) in patients without cardiac ischemia, and found that the most frequently associated conditions were advanced age, decreased renal function, presence of stable coronary artery disease (CAD), hypertension, diabetes and respiratory disorders.

In a subsequent study, Bardají et al. [17] carried out a retrospective analysis of medical data of as many as 1032 patients admitted to the emergency department, for whom at least one HS-cTnI measurement was available. A total number of 681 patients had normal cTnI values at admission (i.e., <URL; 39 ng/L) and were hence excluded from the analysis. Of the remaining 351 patients with cTnI elevations, 139 (40%) were finally diagnosed as having an acute myocardial infarction (AMI), whilst 212 (60%) were found to have other pathological causes sustaining the increased cTn concentration. These included heart failure (29.5%), respiratory infections or chronic obstructive pulmonary disease (COPD; 21.7%), tachyarrhythmia (11.8%), chronic renal failure (CRF; 7.5%), stroke (5.7%), digestive diseases (4.7%) and myocarditis (1.9%). During a follow-up of 12-months, patients with cTn elevation and without a diagnosis of AMI had a similar death rate as those with cTn elevation who have been diagnosed with AMI (hazard ratio (HR), 0.96; 95% CI, 0.54-1.71; p=0.895).

Wu et al. [18] carried out a retrospective study including 6081 subjects aged 50 years or younger, who were admitted with elevated cTn values (either cTnI or cTnT values >URL; many assays were used, but no indication was provided on type and cut-offs) to two large tertiary care centers. The leading cause of cTn elevation was established by reviewing all medical records and the cumulative mortality was ascertained from the a death master file. Overall, 58.8% of these patients received a final diagnosis of AMI, whilst a different diagnosis was made in the remaining 41.2%. These alternative diagnoses included myocarditis (8%), neurological disorders (8%), cardiomyopathies (7%), chronic renal failure (6%), traumatic injuries to the chest wall (5%), pulmonary embolism (4%) and musculoskeletal disorders (4%).

Another observation retrospective study was carried out by González-Del-Hoyo et al. [19], who reviewed the medical record of 3629 patients admitted to the emergency department and for whom at least one HS-cTnI value was available. Overall, 537 patients (14.8%) were found to have a cTnI elevation (i.e., >URL; 40 ng/L,) without referring chest pain. The leading causes of cTnI increase in these patients were heart failure (21.8%), respiratory disorders (16.9%), AMI (7.3%), tachyarrhythmia (6.5%), digestive disorders (4.8%), neurological diseases (4.7%), CRF (3.0%), infectious diseases (2.6%) and pulmonary embolism (1.9%). Notably, the 1-year death rate was neatly 3-fold higher in patients with cTnI elevation and no chest pain than in those with cTn elevation and chest pain (33.9% vs. 14.3%; odds ratio (OR), 3.07; 95% CI, 2.26-4.16).

An interesting analysis has then been published by Campbell et al. [20], who carried out a retrospective 1-year study including 5674 patients in whom a HS-cTnI test was performed at emergency department admission and were then successively hospitalized. Overall, a final diagnosis of AMI could be made in 23.6% of patients, whilst a different diagnosis was made in the remaining 76.4% subjects. An infectious disease (35.4%) was the most frequent extra-cardiac cause of cTnI elevation (defined as a value >URL, i.e., >34 ng/L), followed by neurological disorders (21.3%), respiratory diseases (12.4%), gastrointestinal disorders (8.9%), metabolic disturbances (6.4%), CRF (4.5%), bleeding (2.5%) and malignancies (2.5%).

More recently, AlQassas et al. [21] conducted another retrospective study including 131 patients with HS-cTnT elevation (i.e., >30 ng/L), in whom a cardiac origin could be finally excluded. Infectious diseases (especially urinary tract and respiratory infections) were found to be the leading causes of cTnT elevation (40%), followed by CRF (17%), neurological disorders (11%), and pulmonary embolism (3%).

Lee et al. [22] published the results of an interesting prospective cohort study, in which prevalence, causes and outcome of patients admitted to the emergency department without cardiac ischemia were assessed). Overall, increased HS-cTnI values (>URL; i.e., >34 ng/L in men and >16 ng/L women, respectively) were found in 114/918 (12.4%) patients out of the entire cohort, and the leading underlying pathologies were infectious diseases and/or sepsis (27%), traumas (22%), respiratory diseases (10%), gastrointestinal disorders (6%), CRF (5%) and neurological diseases (3%).

An interesting article has also been published by Korley et al. [23], who carried out a prospective observational study including 808 patients assessed for suspected cardiac ischemia by means of both a traditional (CS) and HS cTnI immunoassays. Overall, 11% patients were found to have normal CS-cTnI (<URL; i.e., <28 ng/L) but concomitantly elevated HS-cTnI (>URL; i.e., >26 ng/L), and the leading underlying pathologies of these subjects were extracardiac diseases (61.5%), followed by acute decompensated HF (25.6%), volume overload of unclear etiology (6.4%), other cardiac pathologies (3.8%) and AMI (2.6%). After a 1-year follow up period patients with both normal values of CS-cTnI and elevated values of HS-cTnI had a nearly double risk of death compared to those with normal values of both CS-cTnI and HS-cTnI (HR, 1.91; 95% CI, 1.14-3.19).

## Causes of death in patients with non-cardiac cTn elevations

Beyond the studies which explored the possible causes of cTn elevation beyond myocardial ischemia, the second essential aspect that shall be addressed is which is the most likely cause of death in a patient with non-cardiac causes of elevation of cTn.

In 2013 Oluleye et al. [24] published the results of a vast prospective study, in which 11,193 subjects aged 54-74 years free of cardiovascular diseases at baseline were followed up for a mean period of ~10 years. Overall, the larger part of subjects with elevated HS-cTnT values (>99^th^ URL, i.e., >14 ng/L) died for cardiovascular diseases (20.6%, composed of 7.4% for AMI, 3.2% for stroke and 10.0% for other cardiovascular conditions), followed by cancer (14.0%), respiratory diseases (4.6%), whilst as many as 60.9% died for other causes which were not specifically described in the published report.

In another article, Dhesi et al. [25] reported the results of a retrospective cohort study including 245 patients hospitalized for extra-cardiac diseases and elevated cTnI values measured with a CS technique (i.e., >150 μg/L, which corresponded to the local URL). Overall, 41.6% of these patients died after a follow-up of 1 year, only 10% of whom for cardiovascular causes. The leading causes of death were respiratory diseases (29.4%), infections (16.7%), neurological disorders (9.8%), cancer (2.0%), metabolic diseases (2.0%) and renal failure (1.0%).

In the retrospective study of Campbell et al. [20], which has been described in the previous section of this article, 5674 patients receiving a HS-cTnI test at emergency department admission were hospitalized and followed up for a 1-year period. Overall, a final diagnosis of AMI could be made in 23.6% of patients, whilst a different diagnosis was made in the remaining subjects. The cumulative mortality at 1 year was over 3-fold higher in patients with extra-cardiac pathologies than in those diagnosed with AMI (35% vs. 13%; OR, 3.59; 95% CI, 2.67-4.84). An infectious disease (35.4%) was the most frequent extra-cardiac cause of cTnI elevation (>URL, i.e., 34 ng/L), followed by neurological disorders (21.3%), respiratory diseases (12.4%), gastrointestinal disorders (8.9%), metabolic disturbances (6.4%), CRF (4.5%), bleeding (2.5%) and malignancies (2.5%).

In the prospective cohort study of Lee et al. [22], 102 patients with increased HS-cTnI values (>URL, i.e., >34 ng/L in men and >16 ng/L in women, respectively) non clearly attributable to CAD, who died during a follow-up of 1 year after emergency departments admission, were investigated. The primary cause of death was sepsis (19.6%), followed by cancer (11.8%), respiratory diseases (6.9%), gastrointestinal disorders (5.9%), cardiac pathologies (4.9%), stroke (2.9%), other vascular diseases (2.9%) and renal failure (2.9%), whilst the reason of death remained almost unknown in 39.2% of all these patients.

More recently, Kaura et al. [26] carried out a large retrospective cohort study consisting of 257,948 consecutive patients who underwent cTn testing (with a vast array of cTnT and cTnI immunoassays) for any clinical reason, followed-up for a mean period of 3.3 years. A total number of 55,850 patients (21.7%) died during follow-up, and the death rate was 3.2-fold more likely in those with elevated baseline cTn values (> assay-specific upper limit of normal; ULN) than in those with cTn concentration <ULN (HR 3.2; 95% CI, 3.1-3.2). Extra-cardiac pathologies were prevailing as primary causes of death (65.2%), with the leading underlying conditions being pneumonia (11.2%), gastrointestinal disorders (7.3%), ischemic heart diseases (7.0%), non-respiratory infections/sepsis (6.6%), cancer (6.4%), stroke (5.7%), atrial fibrillation (4.6%), heart failure (4.2%), musculoskeletal disorders (3.8%), metabolic disorders (3.5%), renal failure (2.8%), neurological diseases (2.7%), cerebral hemorrhage (2.6%), COPD (2.4%) and pulmonary embolism (2.1%).

A very interesting study has then been recently published by Roos et al. [27]. Briefly, the authors performed a prospective study including 19,460 chest pain patients with elevated HS-cTnT values (>URL, i.e., >14 ng/L), who were followed-up during a mean period of 4.0 years. Overall, 1577 patients (8.1%) died during follow-up, 552 of whom for cardiovascular disease (35.0%; composed of 15.2% for AMI, 17.4% for heart failure and 47.6% for other cardiovascular causes) and 65.0% for extra-cardiac pathologies. Among these second cohort of patients, the leading cause of death was cancer (30.8% of all deaths, prevalently composed of 7.2% lung cancer, 4.8% prostate cancer, 2.9% urogenital cancer, 2.7% colorectal cancer, 1.9% hepatic cancer, 1.1% pancreatic cancer, 1.1% breast cancer, 1.1% hematological cancer, 0.3% neurological malignancies). A subanalysis of the entire cohort then revealed that respiratory diseases were the second most frequent causes of mortality (15.7%), followed by neurological disorders (6.6%), digestive diseases (6.4%), infective pathologies (6.1%), psychiatric diseases (4.3%), urogenital disorders (3.5%) and endocrine disturbances (3.5%).

## Discussion

There is now almost incontrovertible evidence that at least one out of twenty subjects undergoing laboratory testing for whatever clinical reason will display a cTn value measured with HS immunoassays exceeding the method-specific URL, and this proportion is especially increased in the male sex, in the elderly, in those with impaired renal function and, especially, in inpatients bearing one or more comorbidities [28]. In such patients limiting the significance of cTn elevation to risk assessment of future CVD only seems reductive, at least for two essential clinical reasons. First, knowing which are the most frequent extra-cardiac causes of cTn increases would guide the clinical reasoning and decision making to providing the best possible care to these patients, i.e., for managing underlying pathologies which may have gone overlooked until an abnormal cTn value has been identified. On the other hand, acknowledging in advance the most likely causes of death in patients with extra-cardiac cTn elevations would also help establishing the most appropriate measures to attenuate the risk of future cardiovascular and non-cardiovascular mortality.

**Figure 1:**
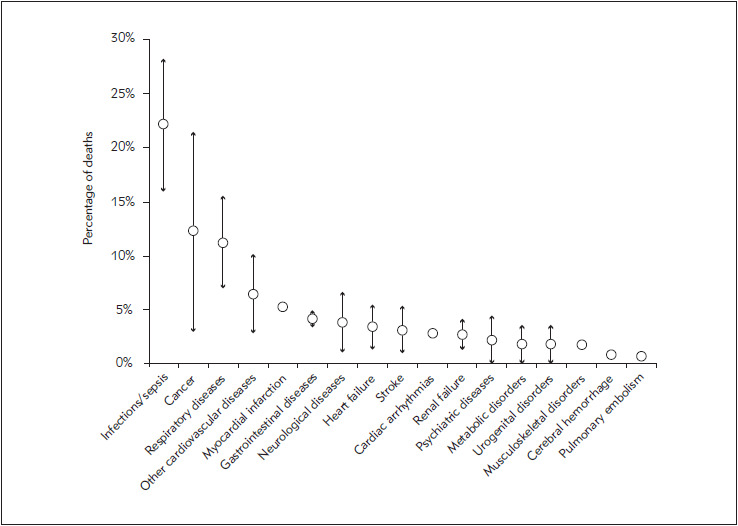
Leading causes of death (mean percent value ± standard deviation of literature data) in patients with extra-cardiac causes of cardiac troponin elevation.

**Figure 2:**
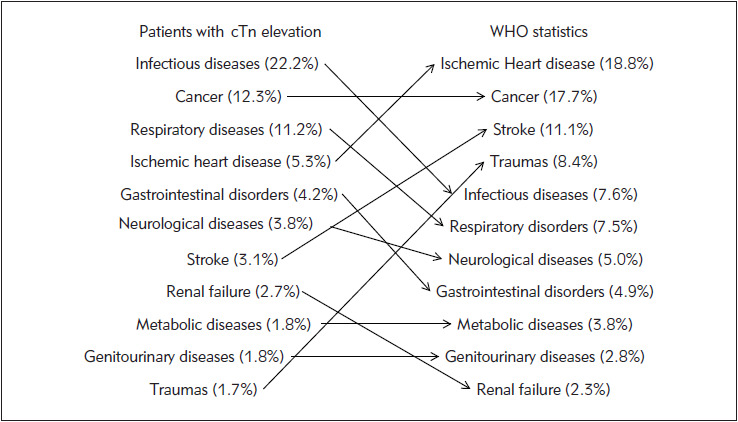
Leading causes of death (mean percent value) in patients with extra-cardiac causes of cardiac troponin elevation compared with those in the general adult population.

Taken together, the current literature data suggests that cTn elevations may be equally common in patients with cardiac and extra-cardiac diseases. Among the latter cohort of patients, the leading extracardiac diseases which may be responsible for either cTnI or cTnT elevations include infectious diseases/ sepsis, pulmonary disorders (e.g., pulmonary embolism, COPD), renal failure, malignancy, as well as gastrointestinal, neurological and musculoskeletal diseases (Table 1). What also emerges rather clearly from the current literature data, is that the risk of dying for extra-cardiac diseases is higher (i.e., between two-to three-fold) in patients with extra-cardiac cTn elevations than in those with cardiac pathologies, and that the most frequent cause of death would then be infections/sepsis, followed by malignancy, respiratory disorders, AMI, gastrointestinal and neurological diseases, heart failure, stroke, cardiac arrhythmias, renal failure, psychiatric, metabolic, urogenital and musculoskeletal disorders (Figure 1). This epidemiologic scenario does not thoughtfully overlaps with the current information on the death rate in the adult general population provided by the World Health Association (WHO) 2016 data on disease burden and mortality estimates [29] (Figure 2). For example, the unexpectedly high burden of mortality for infective diseases in patients with extra-cardiac causes of cTn elevations compared with the current WHO mortality data, combined with the large number of patients in whom that same increase of cTn value is sustained by infections, would lead us to conclude that there is a considerable risk that the underlying pathology causing cardiac injury and cTn elevation would then become the cause of death inthese patients. In keeping with this hypothesis, although ischemic heart diseases is still the leading cause of death worldwide according to recent WHO statistics, it only emerges as the fourth mortality reason in patients with extra-cardiac cTn elevation. Similarly, stroke is the third cause of worldwide deaths according to the WHO data, but appears to be only the seventh cause of death in patients with extra-cardiac causes of cTn elevation.

**Table 1:** Most frequent causes of cTn elevations in the general population according to recent literature data.

Cardiac diseases
Cardiac ischemia (e.g., stable coronary artery disease, acute coronary syndrome)
Congestive heart failure (especially acute decompensated)
Cardiomyopathies
Cardiac arrhythmias (e.g., atrial fibrillation, tachyarrhythmia)
Myocardial infections (i.e., myocarditis)
Chemical injury (e.g., chemotherapy)
Toxic injury (e.g., carbon monoxide poisoning)
Traumatic injury (e.g., commotio cordis)
Extra-cardiac diseases
Infectious diseases/sepsis
Pulmonary disorders (pulmonary embolism, COPD)
Renal failure
Gastrointestinal diseases
Neurological disorders
Musculoskeletal diseases
Malignancy


In conclusion, what emerges clearly from our analysis of the current scientific literature is that cTn elevations may be equally common in patients with cardiac and extra-cardiac diseases, whilst extra-cardiac pathologies then become the leading causes of deaths in these patients. This important evidence shall lead the way to defining appropriate and effective strategies for managing patients with extra-cardiac cTn elevations, so that their risk of future death could be prevented or limited by establishing a more aggressive monitoring and management of the underlying pathologies.

## Conflict of interest statement

The authors declare that they have no conflicts of interest in this work.
